# Mechanical and Microstructural Characterisation of Cooling Pipes for the Compact Muon Solenoid Experiment at CERN

**DOI:** 10.3390/ma14123190

**Published:** 2021-06-09

**Authors:** George Zaburda, Antti Onnela, Kamil Cichy, Jerome Daguin, Alexander J. G. Lunt

**Affiliations:** 1 Department of Mechanical Engineering, University of Bath, North Road, Claverton Down, Bath BA2 7AY, UK; george.zaburda@bath.edu; 2 European Organization for Nuclear Research (CERN), Espl. des Particules 1, 1211 Meyrin, Switzerland; Antti.Onnela@cern.ch (A.O.); kamil.cichy@cern.ch (K.C.); jerome.daguin@cern.ch (J.D.)

**Keywords:** CERN, CMS, microscopy, thin-walled cooling pipes, focused ion beam, digital image correlation, tomography, spectroscopy, profilometry

## Abstract

The Compact Muon Solenoid (CMS) is a particle physics experiment situated on the Large Hadron Collider (LHC) at CERN, Switzerland. The CMS upgrade (planned for 2025) involves installing a new advanced sensor system within the CMS tracker, the centre of the detector closest to the particle collisions. The increased heat load associated with these sensors has required the design of an enhanced cooling system that exploits the latent heat of 40 bar CO_2_. In order to minimise interaction with the incident radiation and improve the detector performance, the cooling pipes within this system need to be thin-walled (~100 μm) and strong enough to withstand these pressures. The purpose of this paper is to analyse the microstructure and mechanical properties of thin-walled cooling pipes currently in use in existing detectors to assess their potential for the tracker upgrade. In total, 22 different pipes were examined, which were composed of CuNi, SS316L, and Ti and were coated with Ni, Cu, and Au. The samples were characterised using computer tomography for 3D structural assessment, focused ion beam ring-core milling for microscale residual stress analysis, optical profilometry for surface roughness, optical microscopy for grain size analysis, and energy dispersive X-ray spectroscopy for elemental analysis. Overall, this examination demonstrated that the Ni- and Cu-coated SS316L tubing was optimal due to a combination of low residual stress (20 MPa axial and 5 MPa hoop absolute), low coating roughness (0.4 μm Ra), minimal elemental diffusion, and a small void fraction (1.4%). This result offers a crucial starting point for the ongoing thin-walled pipe selection, development, and pipe-joining research required for the CMS tracker upgrade, as well as the widespread use of CO_2_ cooling systems in general.

## 1. Introduction

This study was performed as part of the wider development project known as High Luminosity Large Hadron Collider (LHC), which includes the Compact Muon Solenoid (CMS) phase-2 tracker upgrade. The purpose of this upgrade is to allow the LHC to reach an increased beam luminosity of 2500 fb^−1^ by 2025 [1]. To accommodate this increased flux, detector improvements are required at CMS including to the tracker, which is located at the centre of the detector closest to the collision point. Dierlamm A. [2] outlines the new tracker layout, showing how a new type of beam pipe will allow for a more efficient and less costly design.

Currently, CMS uses a 2-phase CO_2_ 2-Phase Accumulator Closed Loop (PACL) cooling cycle within its Pixel Detector, which was installed during its Phase 1 upgrade in 2017 and operates at approximately −33 °C. This type of cooling system is increasingly being used in the thermal management of particle physics experiments including the LHCb Velo, Atlas and the International Space Station’s Alpha Magnetic Spectrometer [3,4,5]. This system is preferred due to its greater heat coefficient and non-toxic, non-flammable CO_2_ coolant that allows for smaller pipes, reduced costs, and a more uniform temperature throughout the cooling system. Moreover, CO_2_ is more environmentally friendly than competing fluorocarbons and has a lower viscosity that makes the distribution and refilling process substantially cheaper [6].

However, numerous technological challenges still exist in reliably implementing these systems, for example the 2-PACL cycle is more suited for smaller systems than those required for the CMS tracker upgrade [7]. Additionally, CMS has added challenges associated with underground space limitations, and a bespoke cooling line system from the surface is currently being designed to overcome this difficulty. These challenges explain why there are no current examples of systems operating at the low temperatures (−40 °C), high pressures (40+ bar), and large heat dissipation loads required by the tracker upgrade [8]. This also explains the importance of taking into consideration the long-term mechanical stability of these systems in order to guarantee continuous reliable use [9].

**Figure 1:**
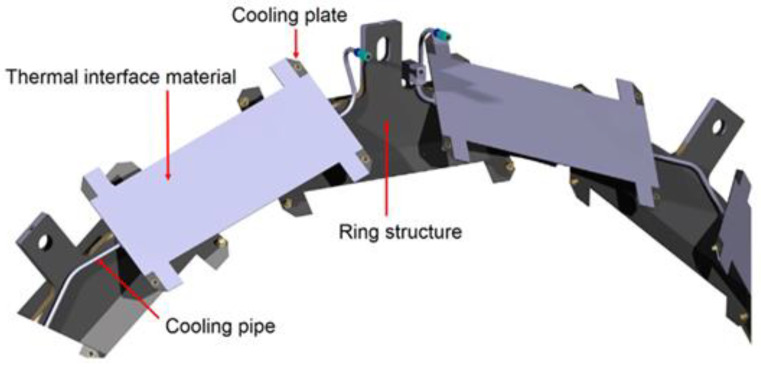
CAD drawing of the TOB showing the complex routing of thin-walled pipes at the CMS [2].

Since the installation of the original CMS tracker in 2007, there has been significant progress in sensor technologies. Consequently, the tracker upgrade will make use of state-of-the-art front-end chips made from a complementary metal-oxide semiconductor [10]. This will allow each chip to identify both a particle’s position and its trajectory. The resulting detector will therefore represent a significant improvement in particle tracking performance but will also require significantly more power than the existing design. Consequently, the heat load of this system will be over 100 kW [11], and the system will require an enhanced cooling system to operate reliably. For this reason, a 40 bar 2-phase CO_2_ cooling system exploiting the latent heat of boiling is currently being designed. Figure 1 shows the design of a typical cooling pipe path mounted on one of the carbon fibre ring structures within the Tracker Outer Barrel (TOB) [2] and the thermal interface material on which the advanced detectors are due to be mounted.

One of the major considerations when designing accelerator physics detectors is signal ‘shadowing’ in which the presence of matter between the collision point and subsequent detectors reduces particle measurement sensitivity at larger distances. For this reason, the mass of all components close to the collision point needs to be minimised. Therefore, when designing the cooling system for the tracker upgrade, pipes of minimal diameter (1.6 to 2.2 mm) and wall thickness (typically <500 μm) have been selected. In order to commence the design and material selection for this system, a comprehensive understanding of the merits and weaknesses of existing cooling pipes is required. Therefore, a sample set of 22 pipes with various material compositions was gathered from previous CMS and ATLAS installations. Historically, these materials have been specifically produced for use in these detector cooling systems and were drawn into pipes several meters long before they were cut to length. During manufacture, they were then bent to the required shape and leak tested before installation. The pressure of cooling systems making use of these thin-walled pipes has previously been 1 to 2 orders of magnitude lower than of those being proposed in future systems (0.1–4 bar, vs. 40–200 bar in future). These relatively low magnitude pressures meant that few experimental studies were performed, and therefore the literature on the mechanical characterisation of this type of thin-walled cooling pipe and/or connection is very limited.

As shown in Figure 1, the thin-walled cooling pipe system will require several detachable connections for testing and to connect the system to the supply/return systems during installation. A mini Vacuum Coupling Radiation (VCR) design has been designed by CERN specifically for this requirement [12]; however, these systems still require the pipes to be permanently joined to brass glands. The most promising approaches for these connections are orbital welding [13], laser welding [14], and brazing [15]. There is a significant amount of refinement and development going into optimising these connections, which will be based on the results of this study. One of the limitations of all of these methods is that they have to be performed offline due to the size of equipment required to produce the joint. The compact nature of the tracker means that there are a number of joints required where space is severely limited. In these positions, the only currently viable option is to solder the pipes to form a connection. This joining mechanism is known to be weaker than the three offline modes, and therefore achieving the required long-term mechanical performance of these types of joints is proving to be a challenge. Significant research is currently being directed towards achieving a solder joint that can withstand the pressures required, and therefore this study contains analysis of these types of connections, as has successfully been demonstrated in previous studies [16,17], in order to form a basis for this subsequent analysis.

The mechanical requirements of these pipes can be used as a basis to perform a material selection exercise of commercially available thin-walled pipes. This analysis revealed three potential wholesale materials with the theoretical performance required for this application; CuNi, stainless steel (SS), and titanium [18,19,20]. In addition to the mechanical characteristics required to maintain the pressure load within the pipes, several other factors need to be considered, including the manufacturability and joinability of these three metals. Whilst all three materials are able to be formed into the shapes required for the CMS cooling system, issues with forming joints between Ti and dissimilar metals are well known [21]. Another factor that requires important consideration of the performance of these pipes is surface roughness, which can induce excessive turbulent flow at roughness greater than 20 μm Ra [22]. One of the other parameters that requires careful consideration and quantification is residual stress. During service, the applied stresses combine with those locked within the structure with the potential to lead to premature failure at loads nominally far below the expected values. Finally, the microstructure and void distribution of the pipes needs to be understood in order to determine the likely impact on the macroscale mechanical performance and leak tightness. For this reason, this study focuses on characterizing these key experimental parameters in existing thin-walled pipes to provide a comprehensive starting point for subsequent optimization of pipe design and joining methods.

## 2. Materials and Methods 

**Figure 2:**
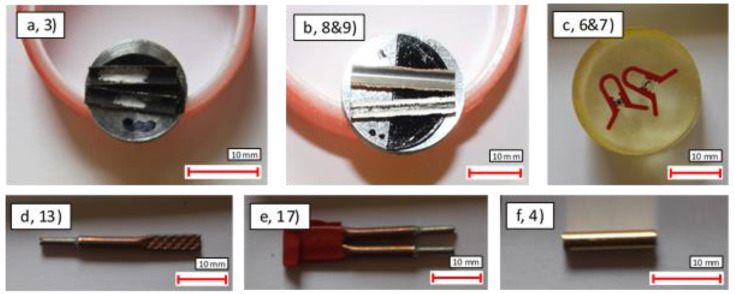
(**a**) Ti + Ni thin-walled pipe (sample 3); (**b**) SS (stainless steel) + Ni + Cu thin and thick-walled pipes (samples 8 and 9); (**c**) SS + Ni impregnated and etched (samples 6 and 7); (**d**) ERSIN joint pipe (sample 13); (**e**) Indium joint pipe (sample 17); (**f**) SS + Ni +Au thin-walled pipe (sample 4).

The 22 pipes investigated represented eight different permutations of cores and coatings: CuNi, SS316L, Ti, SS316L Ni + Au, SS316L Ni, and SS316L Ni + Cu, as shown in Figure 2. These were separated into two categories: sectioned samples and joint pipes. The sectioned samples were chosen to understand the characteristics of the underlying pipes, whereas the joint samples were chosen to understand the effectiveness of existing soldering methods. All experimental analysis was performed at room temperature (20 °C).

To prepare the pipes for analysis, samples of 10 mm length were sectioned using an MTI STX202-A diamond wire saw (MTI Corp, Richmond, CA, USA) with a 2 mm diameter blade, which was operated at a speed of 5 mm/min. To secure the pipes against cutting friction, the pipes were fixed to the cutting bed using wax, which was removed after sectioning using a Fisherbrand FB 15,046 ultrasonic bath of acetone (Fisher Scientific, Waltham, MA, USA).

### 2.1. Sectioned Samples

Twelve out of the twenty-two pipes were sectioned for further investigation using the following naming convention: thick-walled pipes (~0.42 mm wall thickness) and thin-walled pipes (~0.1 mm wall thickness), as shown in Table 1 and Table 2, respectively. Samples 1–3 were uncoated, samples 4–9 were coated with Ni, Au, or Cu, and samples 10–12 were analysed to identify the origin of leaks within this set of materials.

Each of the 22 samples was examined using optical microscopy to assess their structure. In the case of samples 10–12, this process was also used to observe any obvious surface damage that may have been the origin of the leaks. In addition, a selection of samples was characterised using a spectrum of analytical techniques to gain insight into their microstructure and performance. This included scanning electron microscopy (SEM), energy dispersive X-ray spectroscopy (EDX), profilometry, and focused ion beam (FIB) ring-core milling. In order to access the internal surfaces, samples 1–9 were cut axially using the diamond wire saw (Figure 2a,b). To analyse the cross-sectional surfaces, each sample was impregnated into a resin bath (4 g CuSO_4_, 40 mL H_2_O, 20 mL HCl, and 16 g EpoKwick epoxy hardener) and then polished to expose the pipe’s inner cross section using diamond grit (0.5 μm final grade). Microscopy and EDX analysis were then performed on these surfaces before a 1 M HNO_3_ etching solution was applied for 1 hour at room temperature to reveal the grain size and distribution. It should be noted that the cutting and surface preparation parameters used in this study were taken from similar related studies in order to be consistent and produce a representative cross-sectional surface [23].

#### 2.1.1. Optical Microscopy

A Keyence VHX-6000 (Keyence Corp., Osaka, Japan) was used to perform optical microscopy on all of the samples. Imaging was performed at ×100 and ×1000 magnification on the internal, external, and cross-sectional surfaces. In all cases, the Keyence standardized approach was used to obtain an image that was in focus within the view field being examined.

#### 2.1.2. Scanning Electron Microscopy and Energy Dispersive X-ray Spectroscopy 

SEM analysis was performed on samples 1–9 in a JEOL JSM-6480 LV (JEOL Ltd., Tokyo, Japan). Each sample was mounted onto SEM stubs using carbon tape and was placed into a vacuum for 24 h prior to analysis to outgas. A secondary electron detector was used to capture micrographs of each surface at 10 kV. Optimization of the imaging voltage, current, and focal distance was performed using the manufacturer’s recommended approach to achieve high-quality, focused micrographs of each sample.

An Oxford Instruments X-Max detector (Oxford Instruments, High Wycombe, UK) was used to capture EDX points and line scans from each sample. An accelerating voltage of 20 kV was used to generate a spot size of 0.2 μm, which collected data with an increment of 0.25 μm and a dwell of 30 s. These parameters were based on EDX best practice; selection of the accelerating voltage was based on the X-ray absorption energies expected within the samples, and the dwell time was selected to achieve a photon count greater than 100,000 [24]. This analysis was used to determine the chemical composition as a function of position and the elemental makeup of any contaminants. 

#### 2.1.3. Profilometry

Profilometry was performed on the inner and outer surfaces of the pipes using a Scantron Proscan 2000 (Scantron Corp., Eagan, MN, USA). On each surface, three different regions were scanned in order to assess the spatial variation of roughness and, at each point, a map of 1000 × 20 points was collected using a step size of 10 μm and a dwell time of 0.015 s/point. These parameters were obtained following good-practice guidelines in which the number of points and dwell time were increased and the step size was reduced until a consistent roughness estimate was obtained.

#### 2.1.4. FIB Ring-Core Milling for Residual Stress Analysis

Samples 1 to 9 were subjected to residual stress analysis using the FIB milling and Digital Image Correlation (DIC) approach [25,26]. The surface technique allows the near surface (3–10 μm) residual stresses to be quantified and is therefore well suited to analysing the coatings and near surface characteristics of the pipes being analysed in this study. The Tescan Lyra 3 FIB-SEM (Tescan, Brno, Czech Republic) within MBLEM at the University of Oxford was used to repeatedly mill a ring-core feature into the surface of the samples. Milling removes material from the surface and results in localised stress relaxation within the resulting micropillar. Between each milling increment, SEM imaging was performed to record the associated strain relaxation within the micropillar. DIC can then be applied to these images to precisely determine this change in a given orientation. This result can then be compared to the output of finite element simulations of relief within the core, which were originally performed during the development of the ring-core FIB–DIC standard [27]. A comparison with this well-established relief curve allows the determination of the residual stresses that were originally present within the gauge volume.

The experimental technique and parameter used for this study were in accordance with those identified in the ring-core FIB–DIC standard method [27]. Silver paint was applied to each of the mounted samples to increase the strength of the bond and therefore reduce the likelihood of drift during charging. Milling was then performed on the outer surfaces of each of the samples to produce a 5 μm pillar with a 2 μm trench. A FIB current of 250 pA and accelerating voltage of 30 kV was then used to nominally remove 100 nm of material at each milling increment. Tilt-corrected SEM images were recorded between each milling cycle at an imaging voltage of 10 kV and current of 5 nA (which was found to provide optimal brightness and contrast). At each position, 50 images were collected in a time of approximately 45 min. 

To quantify the strain changes within the cores, an opensource DIC package was used [28] in accordance with the standardized approach established and validated by Lunt et al. [17]. Bulk correction was initially performed to accommodate for drift using a reduction factor of 10. Approximately 2000 markers were then distributed across the core, and these 20 × 20 pixel windows were then tracked through all 50 images. The automated outlier removal routine developed by Lunt et al. was used to remove poorly tracked markers [29]. The resultant relief profiles from each sample were then fitted with the finite element ‘master curve’ in order to quantify the full strain relief at infinite milling depth ($\Delta \varepsilon_{\infty }$) in a direction parallel and perpendicular to the pipe axis [27]. The ‘master curve’ is an analytical fit to the generic relief profiles in the pillar, which was first identified by Korsunsky et al. in 2010 [27] and is given by:

(1) $$f\left(\Delta \varepsilon_{\infty },z\right)=1.12\Delta \varepsilon_{\infty }\cdot \frac{z}{1+z}\cdot \left[1+\frac{2}{1+z^{2}}\right],$$

where z=h/0.42d, d is pillar diameter, and h is the milled depth. Given that both h and d are known, curve fitting of this profile can be used to quantify $\Delta \varepsilon_{\infty }$ for a given orientation. The orthotropic nature of the pipe systems being examined in this study meant that strain relief quantification was performed in the two principal axes, namely along the pipe axis (axial) and in a direction perpendicular to the axis of the pipe (hoop). This provides estimates of strain in two perpendicular directions, which can be used to quantify the residual stress tensor, as previously discussed by the authors in detail elsewhere within the literature [30]. In order to perform this conversion of strain relief to estimates of residual stress, the material properties (Poisson’s ratio and Young’s modulus) and associated confidence intervals were required for each of the samples. The values used for this conversion are shown in Table 3. The output of this process was numerical estimates of the residual stress along the pipe direction (axial) and perpendicular to the axis of the pipe (hoop), along with their associated confidence intervals. It should be highlighted that these estimates are an average value for the gauge volume of the ring-core FIB–DIC approach, which is a 5 μm diameter pillar of depth 5 μm. The values are therefore estimates of the near-surface stresses, which is the location of maximum principal stress for pipes produced using drawing, as in this study. Further, the gauge volume for the coated pipes will remain entirely within the coatings (without the substrate), such that the estimates provided are representative only of the coated region.

### 2.2. Joint Samples–Understanding Soldering Connections

The ten jointed samples 13–22 were analysed using X-ray Computer Tomography (CT) in a Nikon XT H 225 ST CT scanner (Nikon Metrology, Bath, UK). The samples were imaged at 64 μA, 134 kV, and 2.83 s exposure and using a 0.25 mm thickness Al filter. An angular step size of 0.1° was used to collect 3600 images of each sample in around 12 h. The specific details of the underlying pipe, coating, type of adapter, and solder of these samples are shown in Table 4. 

It should be noted that the uncoated pipes were soldered using ERSIN 362 flux (Henkel Technologies, Hemel Hampstead, UK) [32], whereas the coated pipes were soldered using no-clean ROL0 (Kester Inc., Itasca, IL, USA) [33]. The reason for this difference is that the wettability of SS surfaces is significantly less than for the coated samples, and a more aggressive flux is required to generate a reliable solder joint. This is problematic in the long-term use of the devices, as aggressive flux promotes joint corrosion over time [34]. For this reason, the coated samples have significant potential in the long-term reliability of the joints, although further micromechanical testing of these joints would be required to maximise the reliability of their long-term use [35,36,37]. It should be noted that samples 20–22 made use of thick-walled pipes (1.6 mm outer diameter), while all others made use of thin-walled (2.2 mm outer diameter).

## 3. Results and Discussion

The results of the samples examined in this study can be split into the two sample type subsets. Characterisation of the pipe microstructure, surface roughness, and residual stress was performed using microscopy, profilometry, and the FIB ring-core approach of the uncoated and coated samples, (Section 3.1 and Section 3.2). In contrast, analysis of the joint samples focused on the effectiveness of the solder connection that was produced when joining the pipes (Section 3.3). An overview of the main considerations has been outlined alongside the results, and the key conclusions are presented in Section 4. 

### 3.1. Uncoated Samples

Analysis of samples 1 to 3 was used to benchmark the behaviour of the underlying pipe characteristics. One of these samples (SS) was selected as the material to coat for the subsequent analysis outlined in Section 3.2.

#### 3.1.1. External Surfaces

**Figure 3:**
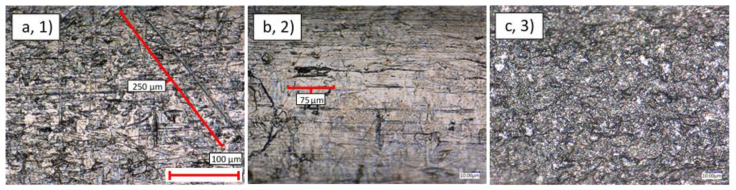
Optical microscopy ×1000 magnification view of the external surface of: (**a**) Cu Ni thin-walled pipe (1); (**b**) SS thick-walled pipe (2); (**c**) Ti Ni thin-walled pipe (3).

**Figure 4:**
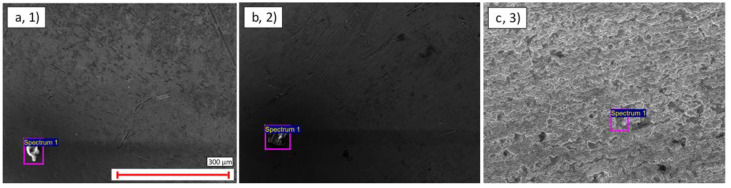
SEM image of contamination on the external surface of: (**a**) Cu Ni thin-walled pipe (1); (**b**) SS thick-walled pipe (2); (**c**) Ti Ni thin-walled pipe (3).

In general, the thin-walled pipes exhibited a smoother and more uniform surface finish than their thick-walled counterparts. Nonetheless, Figure 3 and Figure 4 show examples of scratching, cracking, and contamination observed ubiquitously on samples 1–3. 

To better understand the contaminant characteristics, EDX scans were used to quantify their composition. Overall, as seen in Table 5, it was found that these scans reflected the material composition of the pipes and that C was the predominant contaminant.

**Figure 5:**
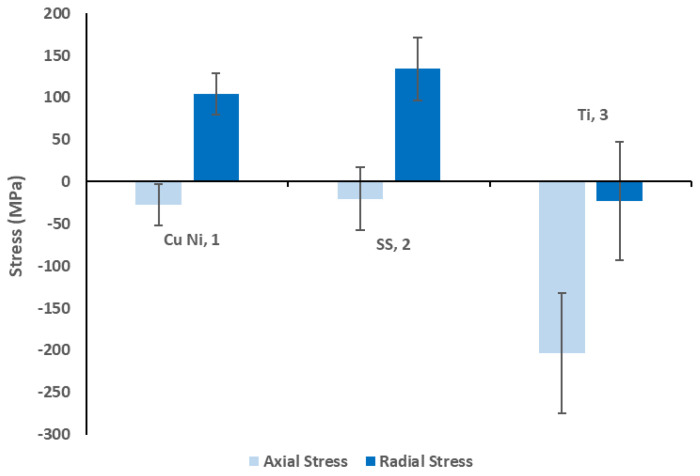
Uncoated samples FIB ring−core analysis results compared to the coated sample mean.

The three samples were also analysed using FIB ring-core milling, and these results can be seen in Figure 5. This showed that the titanium sample exhibited significantly greater axial compressive stresses (200 MPa) than the copper nickel (25 MPa) and stainless-steel samples (15 MPa). In the radial direction, the copper nickel (100 MPa) and stainless steel (125 MPa) samples exhibited significantly greater tensile radial stresses than the titanium nickel sample (20 MPa compressive). Given that the application of pressure to the pipes will induce further tensile forces, this suggests that the residual stresses on the surface of the Ti sample are more suited to high pressure applications. These results have been compared with those obtained from the coated samples in Section 3.2. 

As highlighted in Section 2.1.4, the residual stress estimates obtained through this type of characterisation are highly localised to the first 5 μm of the sample surface. This is the location of maximum principal stress within a drawn pipe [38] and therefore this can be considered to be an upper bound of the stress within the system. It should also be noted that, given that these samples are externally unloaded, the net force generated by the stresses within the cross section must be equal to 0 N. Despite this, an assessment of the average compressive stress within the pipes at the critical Euler buckling load can be determined in order to provide quantitative comparison with the numerical values determined experimentally [39]. In service, the pipes will be simply supported every 10 cm, which corresponds to compressive stresses of 153, 72, and 327 MPa at the Euler buckling load for samples 1 to 3, respectively. This is higher than the 25, 15, and 200 MPa identified within the first 5 μm of the sample surface for these three samples. This demonstrates that even if the entire cross section was subject to this stress value, the load is insufficient to cause buckling. Further, it should be noted that the application of internal pressure to the pipes induces tensile forces which act to oppose buckling. This means that, as has been widely observed in the use of previous thin-walled cooling systems, pipe buckling is unlikely to be of concern during the use of these samples.

#### 3.1.2. Internal Surfaces

**Figure 6:**
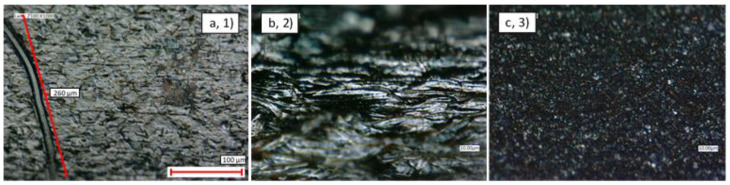
Optical microscopy ×1000 magnification view of the internal surface of: (**a**) Cu Ni thin-walled pipe (1); (**b**) SS thick-walled pipe (2); (**c**) Ti Ni thin-walled pipe (3).

**Figure 7:**
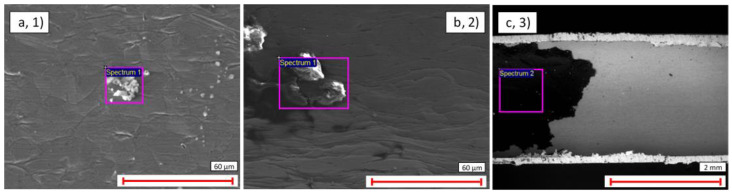
SEM image of contamination on: (**a**) CuNi thin-walled pipe (1); (**b**) SS thick-walled pipe (2); (**c**) Ti with Ni-coated thin-walled pipe (3).

The analysis described in Section 3.1.1 was repeated for the interior surfaces of samples 1–3, and similar levels of scratching, cracking, and contamination were observed as shown in Figure 6 and Figure 7.

However, as before, EDX analysis revealed that the major contaminant observed was carbon, Table 6. Indeed, the significant patch observed on the internal surface of the Ti sample in Figure 7b was found to contain very high levels of carbon (19.8%), and this was the only sample to exhibit this amount of contamination. It is possible that this amount of contamination is anomalous; however, this result does highlight the potential susceptibility of these types of pipes to significant concentrations of internal debris, Table 6. 

**Figure 8:**
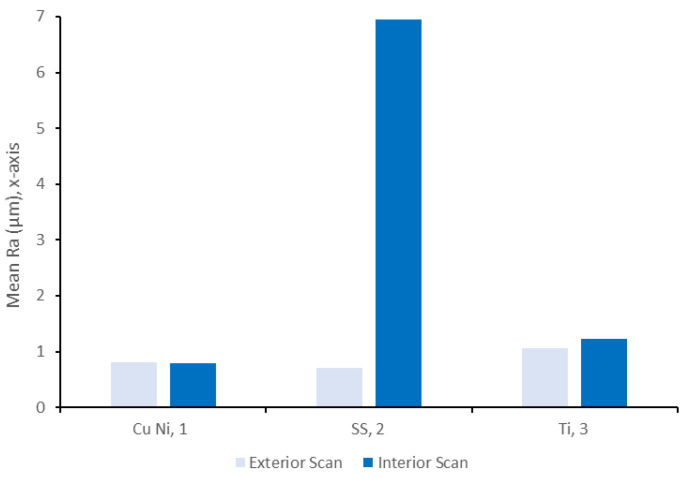
Surface roughness, Ra, values of the interior and exterior surfaces of the uncoated samples compared to the mean of the coated samples.

The profilometry of the interior and exterior surfaces revealed that the interior surfaces roughness was equivalent to or higher than the roughness of the exterior surfaces, as shown in Figure 8. In particular, sample 2 had a mean Ra rate 10 times greater on the interior surface than the exterior surface. Similar results were observed for the coated samples, as outlined in Section 3.2.2.

#### 3.1.3. Cross-Sectional Surfaces

**Figure 9:**
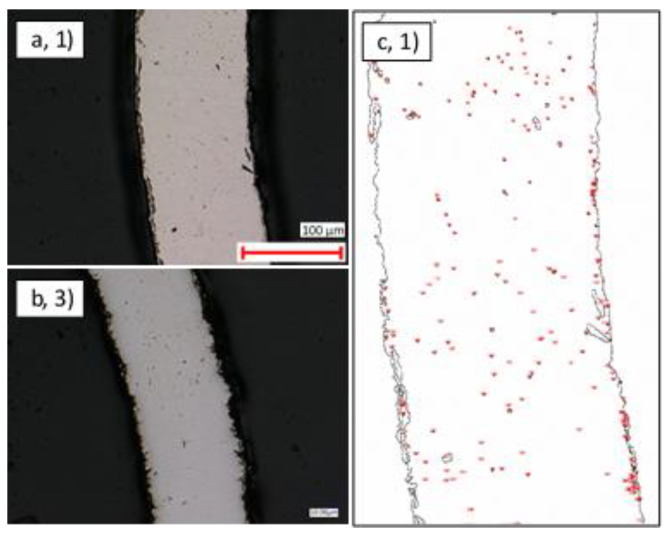
×1000 magnification view of the cross-sectional surface of samples: (**a**) CuNi thin-walled pipe (1); (**b**) Ti thin-walled pipe (3); (**c**) CuNi thin-walled pipe after ImageJ threshold was applied (1).

The analysis presented in Section 3.1.1 and Section 3.1.2 was extended by analysing the structural formations of the cross-sectional surfaces of the three samples. Overall, it was found that each sample had voids within its walls. Analysing these voids using ImageJ’s (Version 1.51, 2018, National Institutes of Health, Maryland, UK) void analysis function, it was observed how the mean diameter and mean area of voids across each sample varied between 0.53–1.32 μm and 0.29–1.58 μm^2^, respectively. These values were found by thresholding a black and white version of each image so that only the voids were considered in the area calculations, Figure 9.

A description of the void areas for each sample have been presented in Table 7, which shows how the normalised total void area per sample varied. Despite the differences in cross sectional area scanned, it can be seen that CuNi contained the largest number of voids at 1.1% of area, SS was an intermediate at 0.24%, and Ti contained the fewest voids at 0.078%. In addition, the mean void diameter and cross-sectional areas were largest in CuNi (1.32 μm and 1.58 μm^2^, respectively) and smallest in the Ti (0.53 μm and 0.33 μm^2^, respectively). This suggests that the CuNi pipes are more affected by porosity during the production process and that Ti pipe production suffers least from the presence of pores.

The influence of internal voids has previously been shown to reduce the local fracture toughness, strength, and ductility in related alloys [40,41,42,43]. However, the impact of these local variations on the global response is highly dependent upon the total void concentration and size. A critical comparison between the results obtained from the three samples suggests that the 1.1% pore content and the 1.32 μm pore size of the CuNi is above the threshold beyond which these local affects begin to detrimentally affect the global response. In contrast, the SS and Ti samples show a reduced concentration (0.24% and 0.078%, respectively) and pore size (0.60 and 0.53 μm, respectively), which is below the threshold at which these affects influence the macroscale behavior. 

The increased pore size and density observed in the CuNi samples are likely to be the origin of the pipe leaking observed and examined in detail in Section 3.1.4. Pipe leak tightness is crucial for the long-term performance of these pipes, and experience has demonstrated that failure through this mode occurs well in advance of catastrophic fracture or yield in the thin-walled pipe designs being considered in this study. Given that the pressure within these systems is consistently monitored and that the leak process naturally acts to reduce pipe loading, the impact of voiding on mechanical properties of these pipes is a secondary criterion, behind leak response.

**Figure 10:**
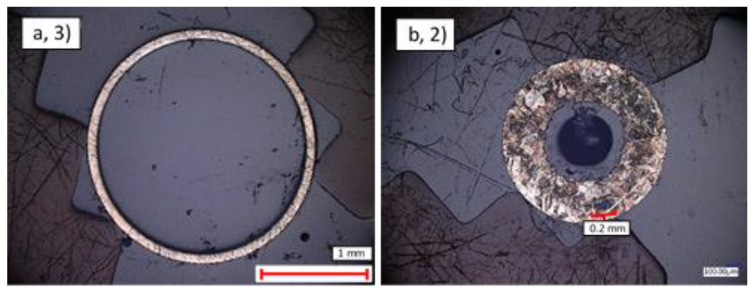
×1000 magnification of the etched cross-sectional surface of samples: (**a**) Ti thin-walled pipe (3); (**b**) SS thick-walled pipe (2).

In order to gain insights into the grain structure, the samples were etched and the resulting grain structures assessed. The contrast within these images was insufficient to perform automated assessment of grain size; however, visual inspection of the cross sections clearly indicated that the grain size within the thick-walled pipes of all samples was in the order of several 10s to 100s of microns. In contrast, the grain size of the thin-walled pipes was below the resolution of the optical microscopes, suggesting that the production process had resulted in a micro-to-nanoscale grain size distribution, as demonstrated in Figure 10.

#### 3.1.4. Leaky Pipes

**Figure 11:**
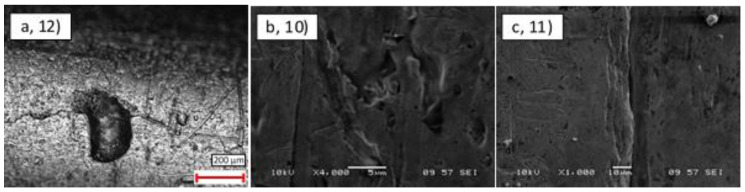
External surface damage on the three leak pipes: (**a**) unknown leak rate (12); (**b**) 3 × 10^−7^ leak rate (10); (**c**) 7 × 10^−4^ leak rate (11).

The leaky CuNi pipes (samples 10–12) were imaged at the locations where leaks had been identified to understand the impact of damage and typical crack sizes. Similar surface features were observed as in the uncoated samples; however, clear craters, cuts, and cracks were discovered on the external surfaces, as shown in Figure 11. This analysis revealed that while in general the roughness and presence of surface pitting/notches in these samples was comparable to the others, they also contained a single larger localised feature that had led to the leak. The type of feature varied from large ‘craters’ of diameter 200 μm to narrow (sub-micron) cracks of up to 150 μm length.

#### 3.1.5. Uncoated Comparison

A comparison between the CuNi, SS, and Ti thin-walled pipes revealed that in general, the exterior surfaces show similar levels of scratching from the production process. Samples 10–12 showed contamination on both the inner and outer surfaces, which is primarily composed of carbon. With the exception of the Ti inner surfaces, these contaminants were microscale and would likely be easily removed using ultrasonic cleaning. The principal outlier in terms of production was the roughness of the internal surface of the SS sample, which was approximately seven times larger than the other two samples. This may suggest that the forming process of this pipe is more challenging; however, the degree of roughness (Ra~7 μm) remains far below the typical Ra = 20 μm required for these systems. In terms of microstructure, all three samples showed significant grain refinement in the thin-walled samples, which likely originates from the increased deformation induced by the forming process. 

A comparison between the mechanical properties reveals that the residual stress within the surface of the Ti samples was more compressive than the other two materials, which indicates an increased resistance to tensile overload. The Ti sample also showed the fewest number of internal voids as well as the smallest average void size, which suggests that this material may be most resistant to localised failure during overload. These results, in combination with the fact that Ti has the highest mechanical strength (600–1250 MPa), lowest density (4.5 gcm^−3^), and smallest coefficient of thermal expansion (8.41 × 10^−6^ K^−1^) of the three options, makes it highly suited for use in cooling pipes. However, at present, a significant challenge remains in the widespread use of this material; effective and reliable joining of this material has not yet been achieved [44]. Therefore, in order to exploit the significant benefits of this material class, further investigations into novel thin-walled pipe joining techniques are warranted.

Of the other two samples, similar magnitudes in residual stress were observed. SS showed reduced numbers of pores when compared to the CuNi, however an increased internal roughness. However, the presence of significant defects in the leaky pipes suggests that there may be issues/challenges in the reliable material class. Given that SS also outperforms CuNi in terms of strength (480 vs. 372 MPa), density (7.87 vs. 8.94 gcm^−3^), and coefficient of thermal expansion (15 × 10^−6^ vs. 16.2 × 10^−6^ K^−1^), this material is preferential to the CuNi. The wettability, weldability, and joinability of SS is significantly higher than Ti, and therefore significant consideration should also be given to this material class.

### 3.2. Coated Samples

In order to understand the potential of coating samples to improve performance and joinability, samples 4–9 were produced using a range of promising coating materials, and the analysis outlined in Section 3.1 was repeated.

#### 3.2.1. External Surfaces

**Figure 12:**
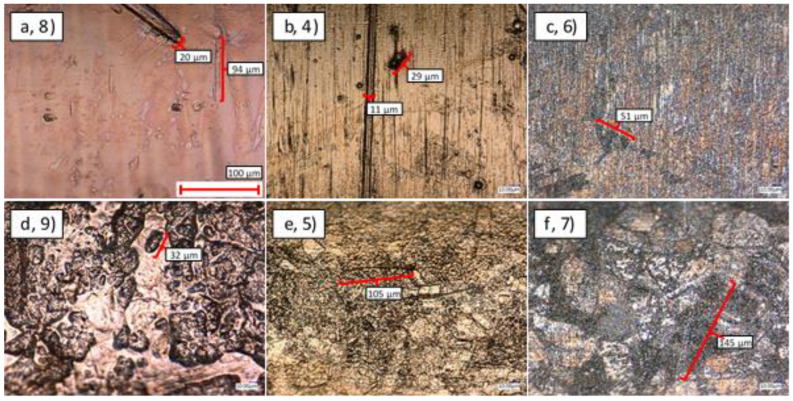
Optical microscopy of the external surface of SS-coated samples: (**a**–**c**) thin-walled pipes, (**d**–**f**) thick-walled pipes, (**a**,**d**) NiCu-coated, (**b**,**e**) NiAu-coated, (**c**,**f**) Ni-coated.

Figure 12 displays the exterior surface quality of thin- and thick-walled coated pipes. In general, the thin-walled pipes exhibited a smoother and more uniform surface finish than their thick-walled counterparts (Figure 12a–c vs. Figure 12d–f). The features highlighted in these figures indicate that although scratches remain on the thin-walled pipes, they are relatively small and widely spaced. In contrast, the thick-walled pipes show roughness across the entire surface. Given the nominally identical coating procedure that was applied to these samples, this suggests that coating is more uniform on samples with a larger outer diameter. 

A comparison between the NiCu-, NiAu-, and Ni-coated thin-walled samples (Figure 12a–c) indicates that the addition of a 4 μm Ni coating is insufficient to hide the grain structure of the underlying pipe. The scratches in the drawn direction are also very clear in the micrograph of this sample (Figure 12c). In the case of Au, the addition of a further 6 μm of material means that the underlying grain structure is no longer visible; however, the axial scratches remain clear. For the Au sample, a further 20 μm of material has been added, which has resulted in a smoother surface where the scratches have been consolidated to leave longer range waviness. 

Similar results can be seen in the case of the thin-walled pipes (Figure 12d–f). In the case of the 4 μm Ni coating, the surface roughness and underlying SS microstructure are clearly visible. The addition of 6 μm of Au is sufficient to hide this underlying structure, but the significant surface roughness remains. The Cu coating has 20 μm of material applied on top of the 4 μm of Ni, which has had the result of smoothing the surface roughness to leave longer range effects over the entire surface.

**Figure 13:**
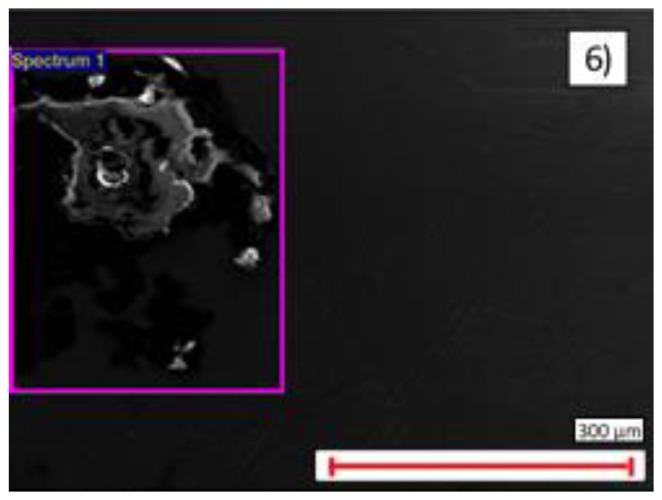
SEM image of contamination on the SS with Ni coating thin-walled pipe (62.7% C, 35.1% Ni, 0.99% Fe, 0.86% O, 0.20% Cr, and 0.13% Al) (6).

SEM imaging of these samples revealed nominally the same level of contamination as their uncoated counterparts, an example of which is shown in Figure 13. Once again, it was found that most of the contamination was C-based, as shown in Table 8. 

**Figure 14:**
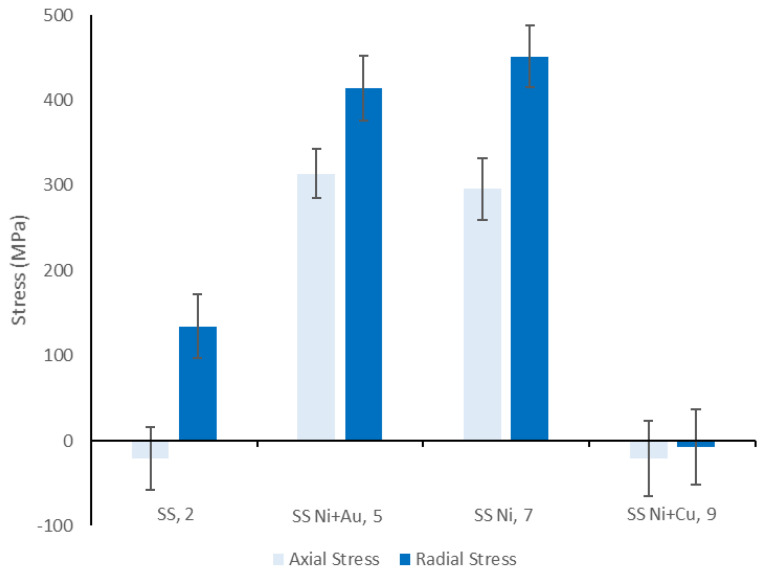
Coated samples FIB ring−core analysis results compared to the uncoated sample mean.

The results of the FIB ring-core milling are shown in Figure 14. Interestingly, this demonstrated that the residual stresses within the Ni- and Ni + Au-coated samples were very similar and significantly more tensile than the residual stresses within the surface of the uncoated SS. In the axial direction, this corresponded to 295 MPa for the Ni and 310 MPa for the Ni + Au, which are around 300 MPa higher than the nominal value in the uncoated SS (15 MPa). In the radial direction, these values are 450 MPa for the Ni and 410 MPa for the Ni + Au, which are once again around 300 MPa higher than the 125 MPa value in the SS uncoated sample. This indicates that the Ni coating process is resulting in a significant thermal expansion mismatch between the substrate and the pipe material and that the Au layer is being nominally stressed to an equivalent value. These tensile stresses are significant, as the coating materials are much closer to their yield strengths, and the addition of forces from the internal pressure within the pipes are likely to lead to coating/interface failure at significantly lower pressures.

Another surprising result was that the residual stresses in the surface of the Cu coating are significantly smaller in magnitude when compared with the Ni, Ni + Au, and underlying SS substrate. This may indicate that residual stresses are reduced by the addition of more material on the surface, or that the deposition process of Cu is gentler, resulting in reduced stresses in the coating. It should, however, be noted that the residual stresses within the Ni layer within this sample are likely to be equivalent to the value measured in sample 7. This means that the sample is likely to fail at this coating layer first, and therefore the entire system is likely to be equivalently resilient to applied stress/pressure as the single coated Ni system. 

**Figure 15:**
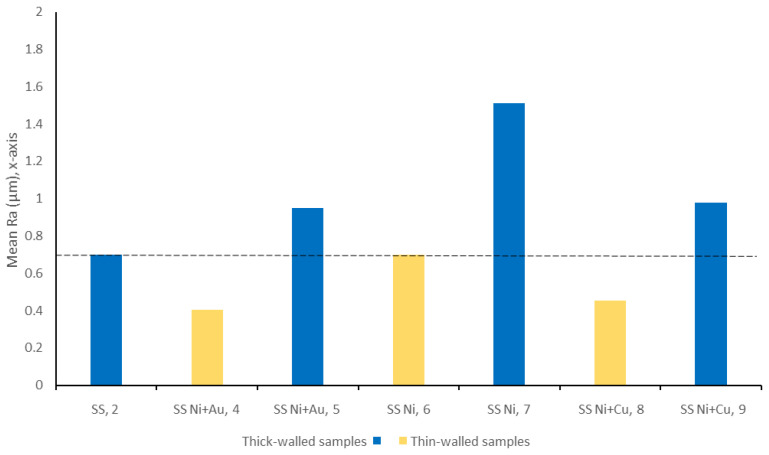
Surface roughness, Ra, values of the exterior surfaces of the coated samples compared to the uncoated samples.

A comparison between the exterior surface roughness of the seven SS samples is shown in Figure 15. The nominal Ra of the uncoated SS surface was 0.7 μm, and therefore it can be seen that the surface roughness of all three of the thick-walled coated samples was rougher than the underlying substrate. In the case of the Ni coating, the thick-walled sample is nearly twice as rough as the uncoated SS, whereas the other two samples show a smaller (20–30%) increase. All of these results correspond well with the images shown in Figure 12. 

In contrast, an examination of the thin-walled pipes indicates that the coating process actually reduced surface roughness or retained approximately the same value. In the case of the 4 μm Ni coating, the roughness was insufficient to induce a significant change in roughness. However, both Au and Cu coatings reduced the Ra value by approximately 50%. Once again, this result compared well with the microscopy results shown in Figure 12.

#### 3.2.2. Cross-Sectional Surfaces

**Figure 16:**
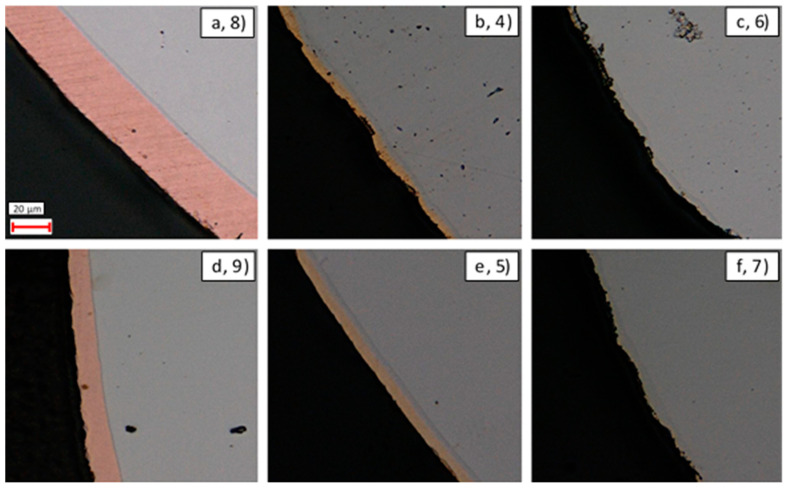
×1000 magnification view of the cross-sectional surface of samples: (**a**–**c**) thin-walled pipes, (**d**–**f**) thick-walled pipes, (**a**,**d**) NiCu-coated, (**b**,**e**) NiAu-coated, (**c**,**f**) Ni-coated.

In order to gain insight into the morphology, elemental diffusion, and thickness of the coatings, cross sectional surfaces of samples 4–9 were prepared, as shown in Figure 16.

It was found that the application of the ImageJ automated void detection procedure (outlined in Section 3.1.3) was ineffective at quantifying the void size distribution within the coatings. This was due to the presence of artificial artefacts that were incorrectly identified as voids and that were found to dominate the numerical estimates obtained. For this reason, qualitative comparisons were instead drawn between the coatings shown in Figure 16 and Table 9.

The worst coating characteristics were observed in the thin-walled Ni-coated sample, number 6, which showed a large number of defects, significant variation in thickness, and a very rough surface. Similarly, the thick-walled Ni-coated sample (equal third) showed a rough surface with a large number of smaller defects, although the coating thickness was more uniform in this sample than in its thin-walled counterpart. 

In general, the Ni + Cu-coated samples (second and equal third) possessed a coating layer that is significantly thicker than the other two samples, with an associated reduction in surface roughness. Despite this, both the thick- and thin-walled Ni + Cu samples contained defects and voids within the cross section. A comparison between the thick- and thin-walled samples revealed that for this set of coating materials, the thick-walled sample possessed an enhanced uniformity, so this sample was ranked higher than its thin-walled equivalent.

Interestingly, the Ni + Au samples showed a significant difference between the characteristics of the thin-walled and thick-walled samples (fifth and first, respectively). The thin-walled sample showed a significant number of relatively large defects, large variations in thickness, and a very rough surface. In contrast, the thick-walled sample had the most optimal characteristics, with no visible voids/defects and a uniform thickness across the sample.

These results demonstrate that quality of coating depends significantly upon the coating material and sample geometry, with no single overriding characteristic being identified across the entire data set. This is indicative of the challenging nature of coating optimisation and highlights that significant focus and optimisation will be required to produce a uniform, defect-free coating that facilitates the enhanced wetting required.

**Figure 17:**
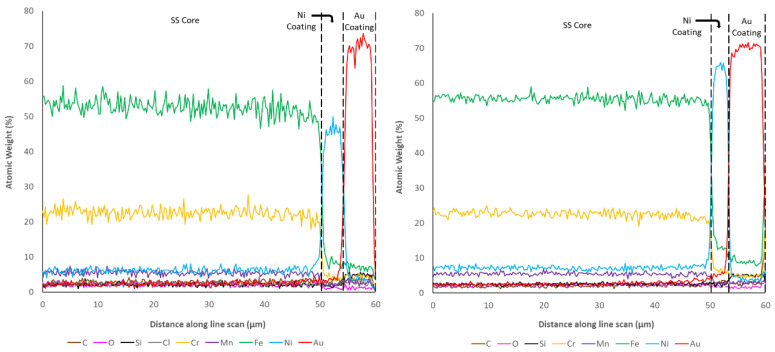
Element atomic weight distribution for the SS Ni + Au thin-walled pipe (**left**, 4) and thick-walled pipe (**right**, 5), at the core-coating boundary.

**Figure 18:**
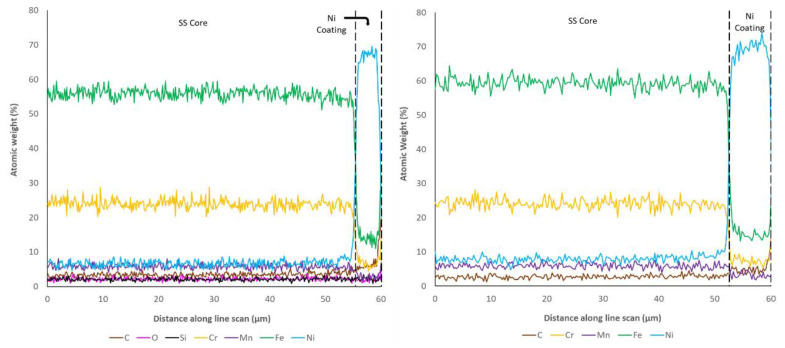
Element atomic weight distribution for the SS Ni thin-walled pipe (**left**, 6) and thick-walled pipe (**right**, 7) at the core-coating boundary.

**Figure 19:**
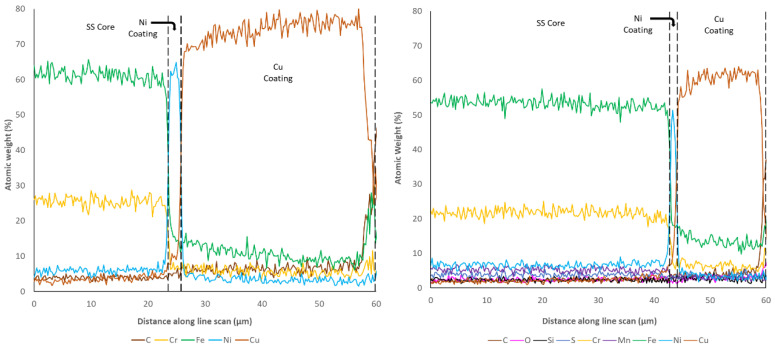
Element atomic weight distribution for the SS Ni + Cu thin-walled pipe (**left**, 8) and thick-walled pipe (**right**, 9) at the core-coating boundary.

Examination of the cross-sectional surfaces also revealed a clearly defined separation of layers in all of the coated samples, which suggested limited diffusion between the substrate and coatings. This was checked through the use of EDX line scans, from the stainless-steel substrate to the edge of the coatings, which are shown in Figure 17, Figure 18 and Figure 19, as previously successfully performed elsewhere [45]. It is important to highlight that despite using a relatively long exposure time per point (30 s), these types of line scan are prone to random noise, and therefore the underlying trends should be the principal focus, rather than the scatter around these trends. In addition, the gauge volume analysed within EDX is bulbous in shape and extends into the sample over a region of a 1–5 microns at 20 kV (depending upon the elements present) [45]. For this reason, sharp transitions become blurred over this characteristic distance.

These studies revealed that the elemental composition of the stainless steel in all samples was close to the nominal distribution expected (~60% Fe, ~20% Cr, etc.). The coatings applied to all samples also clearly showed the highest concentration of the Ni, Au, and Cu in the expected regions. The sharp drops in element concentration between the coatings also suggests that despite making use of a gauge volume of several microns, the interfaces are distinct and the amount of penetration of the coating elements is very limited. However, it can clearly be seen that both Fe and Cr are present at low concentrations (5–10% and 2–5%, respectively) several microns from their nominal coating position. This suggests that atoms from the SS substrate have translated into the thin film coatings during production. Optimisation of the coating procedures would likely be able to minimise this effect.

One of the other parameters that can be checked via the microscopy and EDX line scans of the cross sections is the coating thicknesses of each of the samples. Given that nominally the same coating procedure was applied for the Ni layer on all samples, and the same coating procedure was applied for the thick- and thin-walled samples, a degree of consistency was expected within these results. However, as shown in Table 10, significant inconsistency between the coating thicknesses was observed. Examination of the Ni coating shows that only sample 6, the thin-walled Ni-only coated sample, shows the nominal correct thickness of 4 μm. Sample 7, the thick-walled Ni-only sample, showed a thickness greater than expected (6.86 μm), whereas all other samples that were subsequently coated with Cu or Au were much thinner than expected (<3 μm). This suggests that the subsequent coating procedure may have resulted in the removal of Ni before subsequent film growth occurred. 

Examination of the Au coating thicknesses (5.45 and 6.92 μm) suggests that these are close to the nominally expected 6 μm. In contrast, the expected 20 μm coating of Cu was found to be thinner in the thick-walled sample (15.4 μm) and significantly thicker in the thin-walled sample (31.7 μm). In almost all cases, it was noted that the thickness of the coating on the thin-walled samples was less than the equivalent thick-walled sample, despite the application of the same coating procedure. The only exception to this rule was the thickness of the Cu coating, which was found to be the inverse of this relationship. From an external perspective, the main difference between these two samples is the outer diameter (2.2 mm for thin-walled and 1.59 mm for thick-walled), suggesting that pipe diameter has a significant impact on coating rate. From this, it can be concluded that optimisation of the coating needs to be performed for the final pipe outer diameter in order to generate a coating at the required/expected thickness.

#### 3.2.3. Coated Comparison

A comparison between the six coated samples revealed that, in general, the exterior surfaces show similar levels of scratching from the production process. All six samples showed contamination on both the inner and outer surfaces, which was primarily composed of carbon. However, these contaminants were microscale and would likely be easily removed using ultrasonic cleaning. Overall, the thin-walled samples (4, 6, and 8) had a smoother surface quality both on the interior and exterior surfaces when compared to the thick-walled samples (5, 7, and 9). The principal outlier was the Ni + Au thin-walled sample, which had the smoothest surface quality, approximately five times smoother than the Ni + Au thick-walled sample. Nonetheless, the degree of roughness across each sample (Ra~7 μm) remains far below the typical requirements of Ra = 20 μm required for these systems. 

The Ni + Cu sample had significantly lower residual stresses when compared to the other two sample coatings. However, this may have occurred due to the significantly thicker coating layers. Nevertheless, the greater residual stress values observed in the Ni + Au and Ni coating samples signify that the Ni coating in all samples are likely to be susceptible to early failure when tensile stress is applied.

Examination of the coating microstructures revealed that almost all coatings contained voids and defects. In particular, sample 6, the thin-walled Ni-coated sample, showed the largest number of defects and a highly non-uniform coating. The optimal coating microstructure was in sample 5, the thick-walled Ni + Au-coated sample, which was closely followed by sample 8, the Ni + Cu-coated thin-walled sample. Both of these samples showed very few voids, a consistent coating thickness, and smooth surface. The EDX line scans revealed that, in general, the composition of all samples was expected and the interfaces between the different regions were sharp, but there was significant diffusion of Fe and Cr into all the coatings. In addition, the microscopy of these samples revealed significant variation in coating thicknesses between the samples and distinct differences between the predicted and actual thicknesses. In particular, the external diameter of the pipes was found to play a significant role in the coating rate. This analysis demonstrates that careful optimisation of the coating parameters are required in order to facilitate the reliable use of this method. This will be essential in ensuring that the improved wettability and joinability offered by the coatings is not undermined by ineffective coating strength during service.

### 3.3. Joint Samples–Effectiveness of Coating and Jointing

The principal reason for applying coatings to the ends of the SS samples in this study was to improve the wettability of the surfaces for effective soldering. In order to probe this behaviour, CT was performed on samples 13–22, as listed in Table 4. In the case of samples 13–16, the more aggressive ERSIN solder was required in order to generate a joint between the pipe and the gland. This type of solder is undesirable for long term use, as the corrosive nature of the flux has the potential to lead to joint corrosion and failure during the lifetime of the expected part. However, the addition of coatings improved the wettability of the surface to facilitate the use of ROL0, which makes use of a less-corrosive flux and will therefore have a reduced impact on the long-term performance of the thousands of joints in use within the CMS tracker.

**Figure 20:**
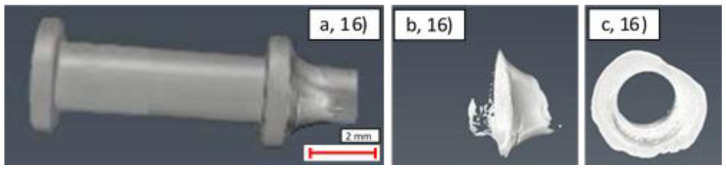
CT images of uncoated sample (16): (**a**) side view of gland exterior; (**b**) side view of interior solder; (**c**) axial view of interior solder.

**Figure 21:**
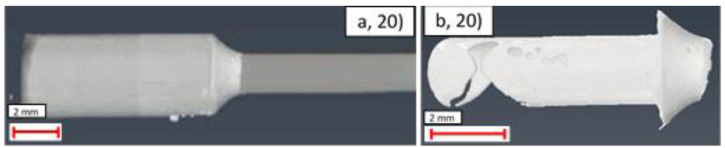
CT images of Ni-coated sample (20): (**a**) side view of pipe and gland exterior; (**b**) side view of left side interior solder.

**Figure 22:**
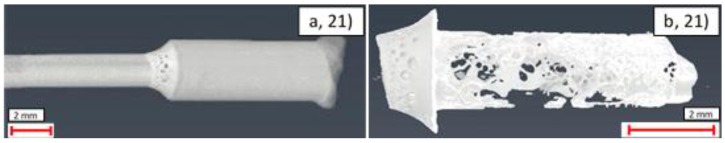
CT images of Ni + Au-coated sample (21): (**a**) side view of pipe and gland exterior; (**b**) side view of right side interior solder.

**Figure 23:**
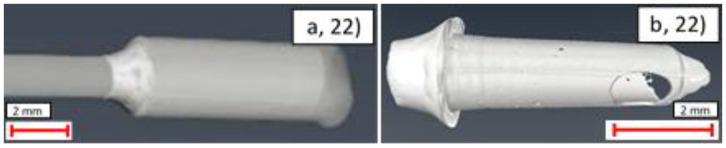
CT images of Ni + Cu-coated sample (22): (**a**) side view of pipe and gland exterior; (**b**) side view of right side interior solder.

Example images from the CT analysis of the representative samples from the uncoated, Ni, Ni + Au, and Ni + Cu samples are shown in Figure 20, Figure 21, Figure 22 and Figure 23. Within these figures, the lower X-ray absorption contrast of the glands and piping means that they appear as darker grey, whereas the higher X-ray absorption contrast of the solder means that it appears as a lighter grey. Segmentation of these differing grey scales allowed the structure of the solder to be revealed in isolation. 

In general, effective soldering is characterised by good penetration of the solder into the region between the gland and the tube. Ideally, the solder within this region should be continuous, and voids/gaps in material should be minimised. In addition, a clear, clean, and continuous fillet should be present at the end of the gland at the position where the solder is applied. Although quantitative measurements can be obtained from CT images, the diverse range of factors revealed through the CT scanning led to challenges in making comparative measurements. For this reason, a qualitative ranking of the key solder characteristics was made, as outlined in Table 11.

An examination of Table 11 reveals that the joint with the worst characteristics is the uncoated sample, number 16, which has incompletely wetted such that the internal solder was almost entirely absent, as shown in Figure 20. Further examination of the ranking demonstrates that in general, despite using the more aggressive ERSIN flux, the uncoated samples were generally the less effective in terms of joint structure (10th, equal 7th, 5th, and 6th), than the coated samples. The exception to this rule is the Ni + Au samples, which also generally showed ineffective joining characteristics (equal 7th and 9th). The characteristic joint type produced in these samples is shown in Figure 22 in which large numbers of voids and limited amount of flux had penetrated into the joint. Operators highlighted that the Au-coated samples appeared to wet very quickly, which may explain why insufficient material had impregnated the gap between the piping and gland.

In contrast to the Ni + Au samples, the Ni and Ni + Cu samples showed an improved joint performance compared to the uncoated samples (1st and 4th, 2nd and 3rd, respectively). In general, these joints showed significant amounts of solder within the joint, an effective shoulder shape, and reduced numbers of voids (both small and large). This reveals that despite making use of the more aggressive ERSIN flux, the uncoated sample solder distribution is generally less effective than that produced by the less corrosive ROL0 flux in these two samples. This suggests that there may be potential to improve solder distribution by coating the ends of the samples. However, as outlined in Section 3.2.3, further optimisation of the coating is required for reliable use of this approach.

Despite these issues, a comparison between the uncoated sample and the coated samples can be performed. This reveals that although the uncoated samples made use of the more aggressive ERSIN flux, the solder distribution in these samples is less effective than that produced by the less corrosive ROL0 flux. This suggests that despite the generally poor quality of all solder joints, there may be potential to improve solder distribution by coating the ends of the samples. However, as outlined in Section 3.2.3, further optimisation of the coating is required for reliable use of this approach.

## 4. Conclusions and Future Work

Analysis of the uncoated Ti, SS, and CuNi substrate piping materials in this study has revealed that all three materials generally exhibit similar levels of roughness and contaminations in their produced forms. However, microscopy of Ti revealed that it had the fewest number of voids and that the stresses within the pipe were most compressive. These characteristics, in combination with core mechanical and thermal properties, mean that this material is likely to be optimum in terms of performance in the CMS tracker upgrade. However, the significant challenges in producing reliable, consistent joints in this material mean that further investigations are required to exploit the advantages of this alloy.

Comparisons between the SS and CuNi substrates revealed similar characteristics in terms of stress and voiding. However, prior experiences with leaking in CuNi pipes and the improved mechanical performance of SS mean that further investigations into SS are warranted. In comparison with Ti, this alloy is significantly easier to join, and this may overcome the limitations associated with this material class for use in the CMS tracker upgrade.

The Ni-, Ni + Au-, and Ni + Cu-coated SS samples examined in this study revealed that in general, the surface roughness is reduced by coating. Despite the potential improved wettability offered by this approach, residual stress analysis demonstrated that the parameters currently being used to coat the Ni led to reasonably high (400–450 MPa) levels of tensile residual stress. The addition of tensile loading forces during use are likely to lead to premature failure of this coating and therefore this highlights that the optimisation of the coating procedures is essential to reliable use. Additionally, the significant variations in the expected coating thickness, the presence of voiding and large variations in coating thickness, as well as diffusion of Fe/Cr from the substrate also highlight potential areas of concern when using this approach. Despite this, of the three coating types studied, the Ni + Cu demonstrated the lowest residual stresses, smoothest surface, a clear interface between the coatings, and the most consistent microstructure, suggesting that this sample has the most potential for use.

CT analysis of soldered samples produced on uncoated, Ni, Ni + Au, and Ni + Cu samples revealed that the soldering procedures currently being implemented are generally ineffective. This leads to the presence of voids, large gaps in the solder, and ineffective fillets. Despite these issues, the application of coatings facilitated the use of a solder making use of a less corrosive flux, which has more potential for long term use in the CMS tracker. The joints within the Ni- and Ni + Au-coated samples were also found to be better than those in the uncoated samples. This suggests that the coating approach has potential for use in future, but that optimisation of the soldering and coating parameters are required to maximise the potential of this method.

The results of this study serve as a crucial starting point for the optimisation of thin-walled pipes and joining methods for the CMS tracker upgrade. In particular, they provide quantitative estimates of the key parameters that affect the structural and cooling performance of these pipes, namely residual stress, roughness, and microstructural properties. The insights form a strong scientific basis for future investigations, which are ongoing. The outcomes presented are likely to be of immediate interest for the accelerator science community. However, with the increasing use of CO_2_ cooling in a broad range of commercial and research sectors, the conclusions presented are also likely to find widespread use in future.

## Figures and Tables

**Table 1:** List of uncoated and coated samples with sample dimensions.

Sample Code	Core	Coating	Inner and Outer Diameter (mm)		Expected Ni Thickness * (μm)	Expected Au/Cu Thickness * (μm)
1	CuNi	-	2.0	2.2	-	-
2	SS316L	-	0.75	1.59	-	-
3	Ti	-	2.4	2.8	-	-
4	SS316L	Ni + Au	2.0	2.2	4	6
5	SS316L	Ni + Au	0.75	1.59		
6	SS316L	Ni	2	2.2		-
7	SS316L	Ni	0.75	1.59		-
8	SS316L	Ni + Cu	2.0	2.2		20
9	SS316L	Ni + Cu	0.75	1.59		

* These are the design parameters for the coating thicknesses. It should be noted that this is an estimate, and the experimental/actual variation from this value will be explored in Section 3.1.3.

**Table 2:** List of leaky pipe samples.

CMS Sample Code	Leak Detection Point	Core	Coating	Inner/Outer Diameter (mm)	
10	Leak at 3 × 10^−3^ mBar/s	CuNi	-	2.0	2.2
11	Leak at 7 × 10^−4^ mBar/s	CuNi	-	2.0	2.2
12	Leak rate unknown	CuNi	-	2.0	2.2

**Table 3:** Material properties used for strain analysis [31].

CMS Sample Code	CES EduPack Material Used	Young’s Modulus (GPa)	Poisson’s Ratio
1	CuNi30Fe1Mn1NbSi	145–150	0.34–0.35
2	X6CrNi25-21	196–204	0.265–0.275
3	R50250	100–105	0.35–0.37
4 and 5	Au	76–81	0.415–0.425
6 and 7	Ni	190–220	0.305
8 and 9	Cu	120–135	0.34–035

**Table 4:** Details of joint samples.

CMS Sample Code	Core	Coating	Adapter (Outer Inner Diameter in mm)	Solder Type
13	SS	-	Cu 3.0/2.2	ERSIN
14		-		ERSIN
15		-		ERSIN
16		-		ERSIN
17		Ni	Cu 3.0/2.2	ROL0
18		Ni + Au		ROL0
19		Ni + Cu		ROL0
20		Ni	Cu 3.0/1.6	ROL0
21		Ni + Au		ROL0
22		Ni + Cu		ROL0

**Table 5:** External surface EDX results of contaminants.

Expected Composition		Actual Composition (%)									
Code	Core	Cu	Au	Ni	Fe	Ti	Cr	P	N	C	Other
1	CuNi	61.5	-	28.9	0.74	-	-	-	-	7.43	1.46
2	SS316L	-	-	10.6	65.0	-	17.5	-	-	2.43	2.38
3	Ti CP2	-	-	-	-	83.2	-	7.57	-	6.25	2.97

**Table 6:** Internal surface EDX compositions from box scans of identified contamination zones.

Expected Composition		Actual Composition (%)									
Code	Core	Cu	Ni	Fe	Ti	Mn	Cr	Si	O	C	Other
1	CuNi	64.9	30.3	0.62	-	0.25	-	-	0.49	3.23	0.23
2	SS316L	-	5.86	59.3	-	3.31	20.5	0.51	-	8.91	1.64
3	Ti CP2	-	-	4.94	44.3	-	-	-	26.0	19.8	5.00

**Table 7:** Void size quantification for the three uncoated samples.

Code	Void Count	Pipe Area Analysed (μm^2^)	Total Void Area (μm^2^)	Total Void Area Normalised (%)	Mean Void Diameter (μm)	Mean Void Area (μm^2^)
1-CuNi	223	30,645	352	1.1	1.32	1.58
2-SS	614	73,289	177	0.24	0.60	0.29
3-Ti	258	108,679	84	0.078	0.53	0.33

**Table 8:** External surface EDX compositions from box scans of identified contamination zones.

Expected Composition			Actual Composition (%)									
Code	Core	Coating	Cu	Au	Ni	Fe	Ti	Cr	P	N	C	Other
4	SS304L	Ni + Au	-	90.3	-	-	-	-	-	3.11	4.14	2.45
5	SS316L	Ni + Au	-	89.9	-	-	-	-	-	1.66	6.84	1.57
6	SS304L	Ni	-	-	93.9	2.03	-	0.42	-	-	3.62	-
7	SS316L	Ni	2.74	-	89.6	2.42	-	0.39	-	-	4.73	0.14
8	SS316L	Ni + Cu	94.8	-	-	-	-	-	-	-	4.83	0.33
9	SS316L	Ni + Cu	93.1	-	-	0.22	-	-	-	-	6.26	0.40

**Table 9:** Qualitative ranking of the joint samples, where 1 represents the optimal characteristics sample (in green) and 6 represents the worst characteristics (in red).

CMS Sample Code	Coating	Void/Defect Number	Void/Defect Size	Thickness Uniformity	Surface Roughness	Overall Ranking
4	Ni + Au	5	5	5	6	5
5	Ni + Au	1	1	1	1	1
6	Ni	6	6	6	5	6
7	Ni	4	2	3	4	=3
8	Ni + Cu	3	3	2	2	2
9	Ni + Cu	2	4	4	3	=3

**Table 10:** List of coated samples comparing measured substrate and coating thickness against nominal thickness.

Sample Code	Expected Ni Thickness (μm)	Measured Ni Thickness (μm)	Expected Au/Cu Thickness (μm)	Measured Au/Cu Thickness (μm)
4	4	2.73	Au-6	5.45
5		3.08		6.92
6		4.03	-	-
7		6.86		-
8		2.57	Cu-20	31.7
9		1.67		15.4

**Table 11:** Qualitative ranking of the joint samples, where 1 represents the optimal characteristics (in green) sample and 10 represents the worst characteristics (in red).

CMS Sample Code	Coating	Shoulder Degradation	Number of Voids	Number of Large Voids	Number of Small Voids	Overall Ranking
13	-	6	6	5	6	6
14	-	5	5	4	5	5
15	-	7	8	7	8	=7
16	-	10	10	10	10	10
17	Ni	3	4	6	3	4
18	Ni + Au	9	9	9	9	9
19	Ni + Cu	4	3	2	4	3
20	Ni	2	1	1	1	1
21	Ni + Au	8	7	8	7	=7
22	Ni + Cu	1	2	3	2	2

## Data Availability

Data are available on request to the corresponding author.
